# The Potential Role of Hamstring Extensibility on Sagittal Pelvic Tilt, Sagittal Spinal Curves and Recurrent Low Back Pain in Team Sports Players: A Gender Perspective Analysis

**DOI:** 10.3390/ijerph18168654

**Published:** 2021-08-16

**Authors:** Antonio Cejudo, Josep María Centenera-Centenera, Fernando Santonja-Medina

**Affiliations:** 1 Department of Physical Activity and Sport, Faculty of Sport Sciences, Regional Campus of International Excellence “Campus Mare Nostrum”, University of Murcia, 30100 Murcia, Spain; 2 Department of Surgery, Traumatology and Orthopedics, Bofill Clinic, 17002 Gerona, Spain; 3 Department of Surgery, Pediatrics, Obstetrics and Gynecology, Faculty of Medicine, Regional Campus of International Excellence “Campus Mare Nostrum”, University of Murcia, 30100 Murcia, Spain; santonja@um.es

**Keywords:** sex-related differences, muscle flexibility, spine–pelvis–leg alignment, injury identification, injury prevention

## Abstract

It is assumed that mechanical restriction of hamstring tightness disrupts sagittal spine–pelvis–leg alignment and alters the lumbar–pelvic rhythm predisposing to low back pain (LBP) in athletes; however, this association is not clear. A prospective cross-sectional cohort study was conducted to determine the influence of hamstring extensibility (HE) on sagittal pelvic tilt, sagittal spinal curves, and LBP in 94 soccer and basketball players (61 man and 33 woman) with (*n* = 36) and without recurrent LBP (*n* = 58). Descriptive analysis displayed significant gender differences for HE, sagittal pelvic tilt, and lumbar curve. Differences were found between the low-HE and high-HE groups in lumbosacral angle in for the maximum trunk forward flexion (LH-MTFP). Low-HE was associated with LH-MTFP, lumbar curve and LBP in male players (*p* ≤ 0.023). In female players, LH-MTFP and lumbar curve were associated with low-HE (*p* ≤ 0.020). Low-HE predicted LH-MTFP (*p* = 0.000; OR = 65.6950) and LBP (*p* = 0.028; OR = 13.915) in male players. The decision tree analysis showed that 50.8% of the players were classified with restricted LH-MTFP, 77.4% with low-HE among male players. The 100% of male players with recurrent LBP had low-HE. The 65% of female players with low-HE had restricted LH-MTFP. Measurement of HE, lumbar curve, and LH-MTFP are important in making training decisions for to reduce the incidence of recurrent LBP in soccer and basketball players.

## 1. Introduction

Low back pain (LBP) is one of the most common health-related complaints in team sport players. Previous systematic reviews have reported that the prevalence of LBP in these athletes ranges from 17% to 94% [1,2]. Specifically, the prevalence of LBP in past 12 months has been reported from 6.5% to 47% in soccer players [3,4,5] and from 12.8% to 44% in basketball players [6,7,8]. A previous episode of LBP is often predictive of future back injury [9], which will affect sports participation and performance [9]. Therefore, prevention of LBP in competitive soccer and basketball players is important to health and sport professionals. Based on assumptions, clinical findings, and scientific studies, various risk factors—such as high body mass [1,10], sports experience [1], muscle weakness [11,12], muscle tightness [13,14,15], sagittal lumbo-pelvic misalignments [12,16], and sagittal spinal misalignments [17,18]—have been postulated as potential predictors of LBP in athletes and general population. In this sense, lumbar hyperlordosis in standing [19,20,21] and lumbar hypolordosis or hypokyphosis-posterior convexity-in trunk forward flexion [22] positions have been associated with LBP. Both spinal misalignments combined with the mechanical overload of repetitive sport-specific movements (trunk flexion, extension, and rotation) result in mechanical stress and/or microtrauma to the different joint tissues, which cause spinal pathologies [23,24,25]. Radiological and magnetic resonance imaging studies in players have shown a correlation between lumbar hyperlordosis and spinal pathologies such as discogenic pathology [25,26,27,28], lumbar disc herniation [27,29], degenerative spine/osteoarthritis [20,28,30], and spondylolysis stress fracture of the pars interarticularis [27,28,30,31]. Most researchers agree that these spinal pathologies and lumbar hyperlordosis are common causes of LBP in basketball [25,26,30,32,33,34] and soccer [23,30,31,32,35] players.

Maintaining of normal sagittal alignment of the spine requires a neutral pelvic position in the major sports postures (standing and trunk forward flexion). Based on the lumbopelvic region, it has been observed that changes in the sagittal pelvic tilt affect the type of lumbar lordosis, hypolordosis or hyperlordosis [36]. The increase in pelvic anteversion causes lumbar hyperlordosis in standing due to iliopsoas tightness [37,38,39], strong trunk extensors and weak trunk flexors [37,40], and gluteus maximus weakness [41]. In addition, trunk extension and flexion are usually accompanied by anterior and posterior pelvic tilt. Lumbar hyperkyphosis is mainly caused by hamstring tightness, which limits anterior pelvic tilt [42,43]. Furthermore, hip and trunk extension are usually also accompanied by anterior and posterior pelvic tilt [36,38]. Posterior pelvic tilt is a result of hip and trunk forward flexion [36,38]. Several authors pointed out that muscle imbalance due to muscle tension and weakness affects neutral lumbopelvic alignment and can cause lower crossed syndrome [44,45], spinal misalignment [37,42,46], and LBP [19,37]. For this reason, several studies have reported that players and non-players with LBP have greater increased anterior pelvic tilt than asymptomatic [38,47,48,49].

Of all the muscles that affect pelvic position, hamstring extensibility (HE) is considered the most important. The hamstring stabilize the pelvis in the sagittal plane by controlling the anterior pelvic tilt during dynamic posture, trunk forward flexion [37,43]. Previous studies have observed that hamstring tightness restricts anterior pelvic tilt in soccer [35,50] and basketball [51] players limiting the range of trunk forward flexion unless compensated for by an increase in lumbar flexion [18,52,53,54]. These studies report that hamstring tightness may play a potential impact on pelvic position, sagittal spinal curves, and LBP in competitive athletes.

On the other hand, gender-related differences in the above risk factors for LBP have been found in the scientific literature. For example, lumbar hyperlordosis predominates in female athletes and thoracic hyperkyphotic in male athletes [55,56,57]. In general, female athletes show better pelvic alignment in static and dynamic postures [56,57,58,59,60,61] than male athletes due to greater HE [62,63,64]. In recent years, a high prevalence of competitive football and basketball players with recurrent LBP problems visiting sports medicine centers has been observed. In order to develop prevention strategies, health and sports professionals are interested in analyzing the connection of risk factors for LBP and understanding how they interact during the sequence of movement of the lumbar–pelvic rhythm, especially in static and dynamic postures. Based on this approach to analysis, the objectives of this study were to determine the influence of HE on sagittal pelvic tilt, sagittal spinal curves, and LBP in soccer and basketball players with and without LBP; and to analyze these interactions between risk factors and LBP from a gender perspective. We hypothesized that HE no limits the anterior pelvic tilt, promote sagittal spinal misalignments, and predisposes to LBP in soccer and basketball players.

## 2. Materials and Methods

### 2.1. Study Design

A current prospective cross-sectional cohort study was conducted to determine the influence of HE on sagittal pelvic tilt, sagittal integral morphotype, and LBP in 94 competitive amateur soccer and basketball players with (*n* = 36) and without LBP (*n* = 58). Familiarization with the testing procedures was conducted during the players’ first visit to the sports medicine center during the pre-competition period. During the second visit, each subject filled out the questionnaire containing demographic, anthropometric, and soccer or basketball training data. In addition, the assessment of HE, sagittal pelvic position, and sagittal spinal curves was performed. LBP-related data were collected over a 12-month period after the assessment session of this study by a principal medical examiner. Players were classified as LBP-free, having recurrent LBP or, having chronic LBP, depending on their LBP history. Between 10 and 12 players per day were assessed by the medical examiners. Each player was examined individually. All players were asymptomatic at the time of assessment.

Participants were instructed to avoid strenuous exercise for 24 h prior to the assessment session. All measurements were taken in a single session held in a private room under standard environmental conditions of 25 ºC, with participants wearing the usual equipment used during training. Players did not perform any warm-up or stretching exercises prior to the testing session. Measurements were taken simultaneously by two consultant traumatologists and orthopedic surgeons with over 30 years’ experience in musculoskeletal assessment. The same lead medical examiner measured sagittal spinal curves and HE. The assisting medical examiner controlled compensatory movements and recorded the data. The order of testing was randomized to eliminate bias that could appear on the results using a specific sequence and each test was performed three times. The average of the nearest measurements was used for further statistical analysis. The data were then analyzed to confirm or reject the null hypothesis described previously.

### 2.2. Participants

A sample of 94 (61 man and 33 woman) competitive amateur players soccer and basketball participated in this study. Their age, height, and weight mass were 24.35 ± 4.76 years (range: 16–30 years), 82.4 ± 11.49 kg (67.3–98.5 kg), and 1.82 ± 0.08 m (1.69–1.95 m), respectively (Table 1). Players’ experience in non-professional leagues was at least three years (8.34 ± 7.51 years) and they trained at least three hours per week (6.52 ± 2.84 h/w). The players had not previously received treatment for frontal or sagittal plane pathology through the use of a brace or specific kinesiotherapy. They did not suffer from symptoms of LBP or musculoskeletal limitations during the assessment session.

This study followed the principles of the Declaration of Helsinki and was approved by the Ethics and Research Committee of the University of Murcia (ID: 1702/2017). Participants were fully informed of the purpose and methodology before the tests and provided signed informed consent. The power of the sample size for this research was analyzed as described in the statistical analysis section.

### 2.3. Self-Administered Questionnaire

The self-administered questionnaire consisted of four major sections for collecting information on demographics, anthropometrics, sports experience, and detailed questions on LBP (location, pain history, and severity). The assistant investigator reviewed the questionnaires information. This examiner assessed the anthropometric data. Players were divided into two groups according to having history of LBP (LBP-group) or not (LBP-free group). Recurrent LBP consisted of episodes of LBP for less than 12 weeks. If LBP lasted longer than 12 weeks, or for at least half the days of the year, it was classified as chronic LBP [65,66].

### 2.4. Assessment of Hamstring Extensibility

**Figure 1:**
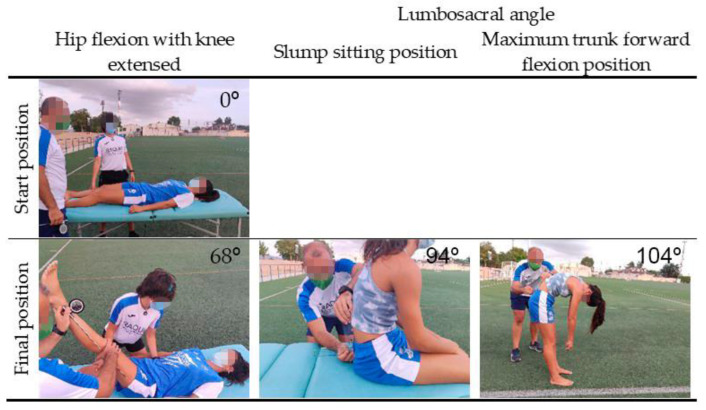
Assessment of hamstring extensibility (HE) and sagittal pelvic tilt.

Maximum passive hip flexion with the knee extended range of motion (HF-KE) for HE was performed using the ROM-SPORT battery methodology according to Cejudo et al. [67]. Both the non-dominant and dominant lower extremities were evaluated. The preferred kicking leg was defined as the ‘dominant’ leg. The HF-KE were measured using an ISOMED Unilevel inclinometer (ISOMED, Inc, Portland, OR, USA). The angle (Figure 1) between the longitudinal axis of the mobilized lower extremity (following its bisector) with the horizontal was assessed [68]. The precision of the inclinometer (ISOMED, Inc, Portland, OR, USA) is two degrees.

### 2.5. Sagittal Pelvic Tilt and Spinal Curves Assessed

**Figure 2:**
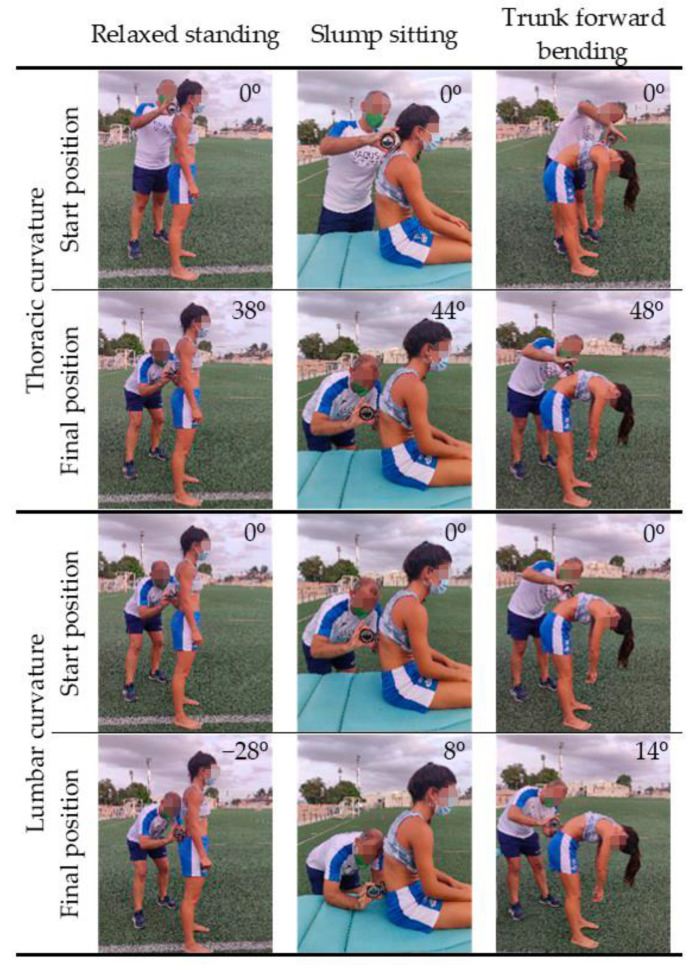
Assessment of sagittal thoracic and lumbar curves.

The lumbosacral or lumbo-horizontal angle (Figure 1) was measured in the slump sitting and in the maximum trunk forward flexion positions according to the methodology described by Santonja et al. [69]. The pelvic position angle was measured with a standard goniometer with level bubble (Baseline, White Plains, NY 10602, USA). Players’ sagittal thoracic and lumbar curves (Figure 2) were evaluated in the relaxed standing, slump sitting, and maximum trunk forward flexion positions as described by Santonja-Medina et al. [69]. The ISOMED Unilevel inclinometer (ISOMED, Inc., Portland, OR, USA) was used to determine the sagittal thoracic and lumbar curves. Lordosis or posterior concavity was recorded with the value having a negative sign, and kyphosis or anterior concavity was recorded with the value having a positive sign.

### 2.6. Statistical Analysis

Data analysis were performed with the SPSS 24.0 software (IBM, Armonk, NY, USA). The *p* value threshold for statistical significance was stablished at 0.05. In order to calculate the power of the sample size, a post hoc power analysis was conducted using the software package G*Power 3.1.9.7. (Heinrich-Heine-Universität Düsseldorf, Düsseldorf, Germany). Normality of data distribution was checked using the Kolmogorov–Smirnov test.

Descriptive statistics were expressed as mean ± standard deviation. Gender differences in descriptive variables were compared using the U de Mann–Whitney test. Dominant and non-dominant HF-KE were compared using Wilcoxon test. The effect size was calculated using the Hedge’s g (95% confidence interval) and was interpreted according to Hopkins et al. [70] as trivial (<0.2), small (0.2 to 0.59), moderate (0.6 to 1.19), large (1.20 to 2.00), very large (2.00 to 3.99), or extremely large (>4.0). Based on the normal ranges of sagittal spinal curves described by Santonja-Medina et al. [69] for the general population, the relative and absolute frequencies with normal spinal alignment or spinal misalignment were calculated. A k-means cluster analysis was performed to determine a cut-off value for HE and to classify players into those with high (high-HE) and low (low-HE) HE. Finally, Pearson’s chi-square test, Cramér’s V test (symmetric or strength of association), and Lambda test (directional or coefficient of predictability/predictive accuracy Guttman’s Lambda) were used to determine the influence of HE (high HE vs. low HE) on sagittal pelvic tilt, sagittal spinal curves, and LBP. According to Lee et al. [71], the interpretation of Cramér’s V and Lambda test was scored as negligible (<0.1), weak (0.1 to 0.3), moderate (0.2 to 0.4), relatively strong (0.4 to 0.6), strong (0.6 to 0.8), and very strong (0.8 to 1.0) association. A binary logistic regression model was used to determine whether low HE predicted restricted sagittal pelvic and spinal misalignment and LBP. In parallel, a decision tree analysis was performed to plot and calculate the probability of pelvic, spinal malalignment, and LBP according to high-HE or low-HF.

## 3. Results

In a previous double-blind study (2 assessment sessions 24 h apart) of 12 young adults, the investigators demonstrated excellent intra-examiner reliability of measurements (sagittal spine curves: ICC ≥ 0.90; DMC_95%_ confidence ≤0.85°; HE: ICC ≥ 0.91; DMC_95%_ confidence ≤5.3°).

Descriptive analysis revealed significant gender differences (Table 1) with moderate effect size for HF-KE (Hedge’s g = 0.95), sagittal pelvic tilt (Hedge’s g = 0.94), thoracic curve in slump sitting position (Hedge’s g = 0.76) and lumbar curve in slump sitting position (Hedges’ g = 0.89). Gender difference showed large effect size for lumbar curve in relaxed standing position (Hedge’s g = −1.53). Sample statistical power was calculated posteriori with input parameters sample sizes of 61 male players and 33 female players, an alpha level of *p* < 0.05, effect size (Hedge’s g = 0.47 to 1.40; Table 1) used for a one-tailed Mann–Whitney U test (G*Power version 3.1.9.7, Heinrich-Heine-Universität Düsseldorf, Düsseldorf, Germany). The variables analyzed obtained a statistical power of ≥0.85 for HF-KE, slump sitting position, LH-maximum trunk forward flexion position, lumbar curve, thoracic curve in slump sitting position, 0.70 for thoracic curve in relaxed standing position, and 0.61 for thoracic curve in MTFP.

For male (Table 2) and female (Table 3) players, differences were found between the low-HE and high-HE groups with very large effect size in HF-KE (male: Hedge’s g = −2.03; female: Hedge’s g = −3.74) and LH-MTFP (male: Hedge’s g = 1.94; female: Hedge’s g = 2.62).

Of all the variables studied, low-HE was significantly associated (*p* ≤ 0.023) with lumbosacral angle in slump sitting position, lumbosacral angle in maximum trunk forward flexion position, lumbar curve and recurrent LBP in male players (Table 4). In female players, lumbosacral angle in maximum trunk forward flexion position and lumbar curve were significantly associated (*p* ≤ 0.020) with low-HE (Table 5).

The recurrent LBP group consisted of 36 players, and the asymptomatic group consisted of 58 players. No player with chronic LBP was identified. Initial stepwise logistic and enter regression analysis revealed that low-EH predicted lumbosacral angle in -maximum trunk forward flexion position (*p* = 0.000; OR = 65.6950; CI_95%_ = 6.806 to 634.122) and recurrent LBP (*p* = 0.028; OR = 13.915; CI_95%_ =1.334 to 145.198) with a high classification accuracy (25 of 61 male players (85.2%)) in male players (sensibility = 85.25%; specificity = 85.30%). In female players, low-HE had no effect on lumbosacral angle in maximum trunk forward flexion position, sagittal spinal curves, and recurrent LBP (*p* ≥ 0.998; OR = 2.059; CI_95%_ = 0.000 to 0.000) with a high classification accuracy (5 of 6 female players (97.00%)).

**Figure 3:**
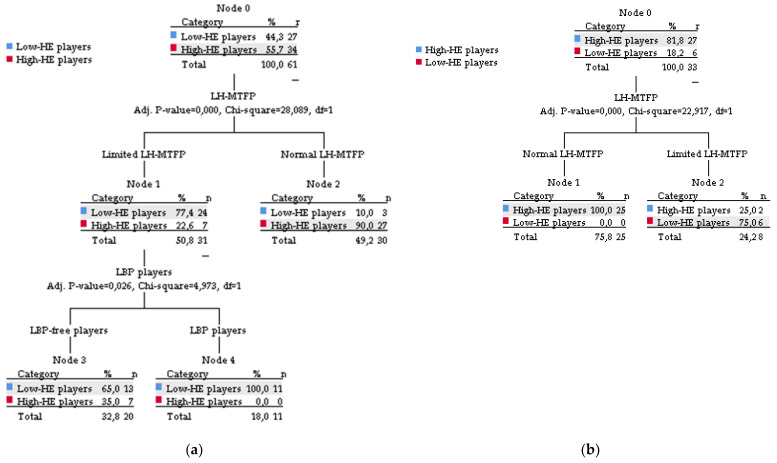
Decision tree analysis of the variables measured in male (**a**) and female (**b**) soccer and basketball players. The cutoff values for male and females were 71° and 75°, respectively.

Finally, among male players (Figure 3), the results of the decision tree analysis showed that 77.4% of the players classified with restricted lumbosacral angle in maximum trunk forward flexion position had low-HE (node 1). Among male players with recurrent LBP (100%), all players had low-HE. The same analysis showed that 65% of female players with low-HE had restricted lumbosacral angle in maximum trunk forward flexion position (Node 4).

## 4. Discussion

To the authors’ knowledge, the present study is the first study to analyze, from a gender perspective, the influence of HE (low-HE versus high-HE) on sagittal pelvic position, sagittal spinal curves and LBP in soccer and basketball players. HE is of particular interest for the prevention and treatment of recurrent LBP. Previous studies have showed the role of hamstring tightness on the spinal injury mechanism and pathogenesis of LBP [15,72]. However, before discussing our finding on this topic, the key point of this article was the gender differences found in most of the variables measured. Therefore, to determine whether HE causes sagittal misalignments of the upper anatomical regions (sagittal pelvic tilt and lumbar and thoracic curves) and recurrent LBP, all variables in this study were analyzed specifically according to gender. It should also be noted in the statistical analysis of this study that previously published values for normality and tightness of hamstring were not used. The main reason for this is the controversial results in the scientific literature, which may be due to different quantitative concepts of hamstring tightness or even the method used to assess muscle extensibility [67]. Female players were found to have a higher HF-KE cut-off than male players. This finding was previously reported in soccer [63,73,74,75] and basketball [6,74,75,76] players. These findings may be explained by the gender differences, including differences in anatomy, such as percentage of muscle mass, sexual dimorphism of the pelvis architecture, lower limbs length, and lower center of gravity [36,77,78], hormonal effects [77], muscle properties, such as muscle stiffness [79,80,81], and fundamental recruitment patterns include walking, bending, and reaching [82,83]. Specific to the spine, gender differences were found in factors related to trunk muscle loads [84,85] and spine–pelvis–leg movement patterns [82]. In addition, a greater habit of flexibility training in female athletes contributes to higher values of muscle extensibility [86]. Therefore, the different values of HE between male and female athletes may show a different influence on pelvic position, sagittal spinal curves, and recurrent LBP (Table 4 and Table 5).

The significant differences between the low-HE and high-HE groups for the lumbosacral angle in maximum trunk forward flexion position provide further evidence that HE influences anterior pelvic flexion during the maximum trunk forward flexion position. Frequency distribution analysis showed that 77.4% and 75% of male and female players with restricted slump sitting position, respectively, had low-HE (Figure 3). The 55.8% of male players with slump sitting position also had low-HE. These results confirm previously published results in soccer [35,50] and basketball [51] players, who had a higher anterior pelvic tilt angle during dynamic posture compared to non-athletes. Trunk forward flexion is the basic posture of sports technical movements that results from coordinated activity between the erector spinae, gluteus maximum, and hamstring [87]. This movement—which combines lumbar flexion, anterior pelvic tilt, and hip flexion—is called the lumbar–pelvic rhythm [43,87]. Considering that the lumbar–pelvic rhythm is necessary for optimal trunk forward flexion, hamstring tightness can result in compensatory movements of the lumbar and thoracic spine to adequately perform the technically required sports movements [16,88]. These altered movement patterns and compensatory movements lead to excessive mechanical stress and strain on the lumbar tissues, which promotes spinal injuries and LBP [18,43,88,89]. For this reason, it is normal to find a high incidence of hamstring tightness in the population with LBP [16,49]. In relation to the results of slump sitting position, several research studies have also shown that the HE does not significantly affect the pelvic tilt in the static position such as standing or sitting [16,19,52,53,90,91].

A second finding of this study was that the low-HE group was significantly associated with lumbar curve in male and female players (Table 4 and Table 5). This is the first study to report that lumbar spinal curve is influenced by low-HE values in soccer and basketball players. Of the 18 male players classified with lumbar sagittal misalignment, 66.7% had low-HE (Figure 3). The percentage was lower in female players (42.1%). However, in the descriptive analysis, it should be highlighted that low-HE group was composed of only eight female players. The results of this study are consistent with previous studies reporting that low-HE was associated with lumbar sagittal misalignments in different sports. Rodríguez-García et al. [92] found correlation values between HE in relation to thoracic, lumbar, and pelvic curves in 243 athletes. López-Miñarro and Alacid [54] and López-Miñarro et al. [93] found greater lumbar flexion and posterior pelvic tilt in kayakers and canoeists with lower HE. A high rate of equestrian riders with lumbar spinal misalignments (functional lumbar hyperkyphosis, hyperlordotic posture, and lumbar hypermobility) and hamstring tightness was also found [56]. Recently, Sainz de Baranda et al. [62] found a relative frequency of 84% hamstring tightness and 66.2% functional lumbar hyperkyphosis in 74 inline field hockey players. In contrast, hamstring tightness did not cause lumbar sagittal misalignments in rowers [94] and young athletes [95]. However, we should not forget that other factors such as weakness of the abdominal muscles [40,96]; and gluteal muscles [41], tightness of the hip flexors [37,38,39], and sagittal pelvic misalignment [36] also determine the alignment of the lumbar curve or not.

A final finding of this study was that low-HE was significantly associated with recurrent LBP only in male players (Figure 3). Mechanical restriction of hamstring tightness disrupts normal sagittal spine–pelvis–leg alignment [18,42,43] and alters the sequence of movement of the lumbar–pelvic rhythm, especially in dynamic postures such as maximum trunk forward flexion position [16,87,88]. This can lead to excessive lumbar tissues loading [97] and lumbar intradiscal pressure [98,99,100], predisposing individuals to LBP [43,98,100]. This biomechanical movements sequence was observed in the decision tree analysis results with the exception of the sagittal lumbar curve in male players. This analysis revealed that 77.4% of players with restricted lumbosacral angle in maximum trunk forward flexion position and 65% of players with recurrent LBP had low-HE. Our results in male soccer and basketball players are similar to previous association and correlation reports. Recently, a systematic review with meta-analysis concluded that individuals with LBP in the general population HE and stiffness are impaired [101]. Significant limitation in HF-KE range of motion and HE in the LBP group compared with the control group is consistent with previously reported findings [15,70]. Furthermore, Radwan et al. [102] demonstrated that the greater the hamstring tightness experienced by the patient, the greater the severity of LBP.

In contrast, no significant association was found between lumbar curve and recurrent LBP in female players (Figure 3). This result has been previously reported by other authors in athletes and non-athletes. Nadler et al. [103] showed no association between HE or leg length discrepancy, and the development of LBP in athletes of different sports including soccer and basketball. The study of Stutchfield and Coleman [94] reported that LBP was not associated with HE in male university rowers. Active adults with LBP had significantly shorter HE than the asymptomatic ones [42,104]. In female players, LBP is possibly associated with other maladaptive postural strategies of regular sports practice [18,53,72] caused by restricted lumbosacral angle in maximum trunk forward flexion position and low-HE.

Further studies are required to determine the influence of hamstring tightness on the observed sagittal movement patterns (spine–pelvis–leg alignment) and lumbar–pelvic rhythm. This strategy will aid in the design of a stretching program that includes stretching exercises that comprehensively train sagittal movement patterns and lumbar–pelvic rhythm in athletes with and without LBP. Measurement of HF-KE, lumbar curve, and lumbosacral angle in maximum trunk forward flexion position are important in making training decisions for to reduce the incidence of LBP in soccer and basketball players. Moreover, this study should increase the sample size. In the case of this study, the sample size of the players, especially female players with low hamstring extensibility and limited anterior pelvic flexion, was limited. Increasing the sample size will help to counterbalance the number of participants in both categories for all variables evaluated in order to decrease the error in the identification of risk factors and prediction of LBP.

## 5. Conclusions

Gender differences were found in sagittal pelvic position, thoracic curve in slump sitting position, lumbar curve in relaxed standing position, and slump sitting position. In male and female players, differences were found between low-HE and high-HE groups in HF-KE and lumbosacral angle in maximum trunk forward flexion position. The probability of low-HE influences on the pelvis is 77.4% in male players with restricted lumbosacral angle in maximum trunk forward flexion position, and 100% in recurrent LBP players. For female players, the probability of low-HE influences on the pelvis is 75% in players with restricted lumbosacral angle in maximum trunk forward flexion position.

## Figures and Tables

**Table 1:** Data related to hamstring extensibility, sagittal pelvic position, and sagittal spinal curves in soccer and basketball players according to gender.

Variables (Degrees)		Male (*n* = 61)	Female (*n* = 33)	*p*-Value	Effect Size Hedge’s g	Total ^1^ (*n* = 94)
HF-KE		70.71 ± 12.16	82.67 ± 12.84	0.000	Moderate (g = −0.95)	74.91 ± 13.16 *
Pelvic position	LH-SSP	103.43 ± 7.89	95.45 ± 9.17	0.000	Moderate (g = 0.94)	100.63 ± 9.15 *
	LH-MTFP	100.26 ± 13.44	86.64 ± 15.70	0.000	Moderate (g = 0.94)	95.48 ± 15.62 *
Thoracic curve	RSP	49.23 ± 8.15	44.94 ± 10.09	0.052	Small (g = 0.47)	47.72 ± 9.07
	SSP	51.92 ± 9.48	44.55 ± 9.76	0.001	Moderate (g = 0.76)	49.33 ± 10.16 *
	MTFP	73.25 ± 9.87	68.97 ± 9.90	0.061	Small (g = 0.42)	71.74 ± 10.04
Lumbar curve	RSP	−32.51 ± 7.27	−43.33 ± 8.24	0.000	Large (g = −1.40)	36.30 ± 9.20 *
	SSP	9.79 ± 8.09	1.55 ± 11.01	0.000	Moderate (g = 0.88)	6.89 ± 9.98 *
	MTFP	17.49 ± 6.62	12.94 ± 9.43	0.018	Small (g = 0.58)	15.89 ± 7.98

^1^ Total: mean ± standard deviation male and female; * Significant differences and moderate or larger effect sizes in the variables measured according to gender; HF-KE: Hip flexion with the knee extended range of motion; RSP: relaxed standing position; SSP: slump sitting position; MTFP: maximum trunk forward flexion position; LH-SSP: lumbosacral angle in slump sitting position; LH- MTFP: lumbosacral angle in maximum trunk forward flexion position.

**Table 2:** Comparative analysis between low (*n* = 27) and high (*n* = 34) hamstring extensibility groups on sagittal pelvis position and spinal curve in male in soccer and basketball players.

Variables (Degrees)		Low-HE (<71°)	High-HE (≥71°)	*p*-Value	Effect Size Hedge’s g
HF-KE		60.96 ± 7.47	78.46 ± 9.23	0.000	Very large (g = −2.03) *
Pelvic position	LH-SSP	105.44 ± 6.05	101.82 ± 8.85	0.153	Small (g = 0.46)
	LH-MTFP	110.81 ± 7.80	91.88 ± 10.81	0.000	Large (g = 1.94) *
Thoracic curve	RSP	49.00 ± 8.37	49.41 ± 8.10	0.615	Trivial (g = −0.04)
	SSP	52.93 ± 10.05	51.12 ± 9.07	0.448	Trivial (g = 0.18)
	MTFP	75.07 ± 10.68	71.79 ± 9.07	0.107	Trivial (g = 0.33)
Lumbar curve	RSP	−31.52 ± 8.86	−33.26 ± 5.74	0.211	Trivial (g = −0.23)
	SSP	11.37 ± 6.45	8.53 ± 9.08	0.186	Trivial (g = 0.34)
	MTFP	18.41 ± 6.12	16.76 ± 6.99	0.718	Trivial (g = 0.24)

* Significant differences and moderate or greater effect size in the variables measured according to the classification of hamstring extensibility. Low-HE: Low hamstring extensibility; High-HE: High hamstring extensibility; HF-KE: Hip flexion with the knee extended range of motion; RSP: relaxed standing position; SSP: slump sitting position; MTFP: maximum trunk forward flexion position; LH-SSP: lumbosacral angle in slump sitting position; LH-MTFP: lumbosacral angle in maximum trunk forward flexion position.

**Table 3:** Comparative analysis between low (*n* = 8) and high (*n* = 27) hamstring extensibility groups on sagittal pelvis position and spinal curve in female soccer and basketball players.

Variables (Degrees)		Low-HE (<75°)	High-HE (≥75°)	*p*-Value	Effect Size Hedge’s g
**HF-KE**		61.50–4.80	87.37–8.50	0.000	Very large (d = −3.74) *
Pelvic position	LH-SSP	98.17–7.81	94.85–9.47	0.508	Small (d = 0.35)
	LH-MTFP	110.33–7.00	81.37–11.65	0.000	Very large (d = 2.62) *
Thoracic curve	RSP	45.83–9.97	44.77–10.30	0.838	Trivial (d = 0.10)
	SSP	49.33–9.44	43.48–9.67	0.205	Moderate (d = 0.60)
	MTFP	74.33–8.33	67.78–9.96	0.145	Moderate (d = 0.66)
Lumbar curve	RSP	−46.33–11.48	−42.67–7.46	0.424	Small (d = 0.41)
	SSP	0.01–16.83	1.89–9.70	0.946	Trivial (d = 0.15)
	MTFP	6.00–15.07	14.48–7.23	0.158	Moderate (d = −0.86)

* Significant differences and moderate or greater effect size in the variables measured according to the classification of hamstring extensibility. Low-HE: Low hamstring extensibility; High-HE: High hamstring extensibility; HF-KE: Hip flexion with the knee extended; RSP: relaxed standing position; SSP: slump sitting position; MTFP: maximum trunk forward flexion position; LH-SSP: lumbosacral angle in slump sitting position; LH- MTFP: lumbosacral angle in maximum trunk forward flexion position.

**Table 4:** Variables associated (expected frequency greater than 5) with high or low hamstring extensibility in male soccer and basketball players.

Variables		Low-HE (≤71°)	High-HE (>71°)	Chi-Squared Test (ꭕ^2^)	*p*-Value	Cramér’s V	Guttman’s Lambda
LH-SSP *	Normal	3 (16.7%)	15 (83.3%)	7.882	0.005	Moderate 0.359	Weak 0.185
	Restricted	24 (55.8%)	19 (44.2%)				
LH-MTFP *	Normal	3 (10%)	27 (90%)	28.089	0.000	Strong 0.679	Strong 0.630
	Restricted	24 (77.4%)	7 (22.6%)				
Lumbar curve *	Normal	15 (34.9%)	28 (65.1%)	5.195	0.023	Moderate 0.292	0.222 Weak
	Spinal misalignment	12 (66.7%)	6 (33.3%)				
LBP *	LBP-free	14 (32.6%)	29 (67.4%)	8.091	0.004	Moderate 0.364	0.296 Weak
	Recurrent LBP	13 (72.2%)	5 (27.8%)				

* Variables significantly associated with high or low hamstring extensibility; Low-HE: Low hamstring extensibility; High-HE: High hamstring extensibility; LH-SSP: lumbosacral angle in slump sitting position; LH-MTFP: lumbosacral angle in maximum trunk forward flexion position.

**Table 5:** Variables associated (expected frequency greater than 5) with high or low hamstring extensibility in female soccer and basketball players.

Variables		Low-HE (≤75°)	High-HE (>75°)	Chi-Squared Test (ꭕ^2^)	*p*-Value	Cramér’s V	Guttman’s Lambda
LH-MTFP *	Normal	0 (0%)	25 (100%)	22.917	0.000	Strong 0.667	Strong 0.833
	Restricted	6 (75%)	2 (25%)				
Lumbar curve *	Normal	0 (0%)	14 (100%)	5.404	0.020	Relatively strong 0.405	0.000 Weak
	Spinal misalignment	8 (42.1%)	11 (57.9%)				

* Variables significantly associated with high or low hamstring extensibility; Low-HE: low hamstring extensibility; High-HE: high hamstring extensibility; LH-MTFP: lumbosacral angle in maximum trunk forward flexion position.

## Data Availability

The data sets used and analyzed during the current study are available from the first or corresponding author on reasonable request.
